# The association of young age with local recurrence in women with early-stage breast cancer after breast-conserving therapy: a meta-analysis

**DOI:** 10.1038/s41598-017-10729-9

**Published:** 2017-09-11

**Authors:** Xiang-Ming He, De-Hong Zou

**Affiliations:** Department of Breast Surgery, Zhejiang Cancer Hospital, Hangzhou, China

## Abstract

The aim of this meta-analysis is to determine the relationship between young age and local recurrence in patients with early-stage breast cancer after breast-conserving therapy. Eligible studies were retrieved from various electronic databases. Among the 19 studies included, 14 studies were analyzed for 5-year local recurrence rate and 8 studies for 10-year local recurrence rate using random effects models. Both results showed that young patients were at higher risk of local recurrence compared to old patients (5-year: RR = 2.64, 95% CI (1.94–3.60); 10-year: RR = 2.37, 95% CI (1.57–3.58)). Harbord’s modified test showed the presence of publication bias in both 5- and 10-year local recurrence rates (P = 0.019 and P = 0.01, respectively). While the Trim and Fill analysis showed that the presence of publication bias did not affect the overall outcome of the 5-year local recurrence rate (RR = 2.21, 95% CI (1.62, 3.02)), it significantly affected the effect size of the 10-year local recurrence rate (RR = 1.47, 95% CI (0.96, 2.27)). Young age is a significant risk factor for local recurrence developed within 5 years of breast-conserving therapy in patients with early-stage breast cancer. Further high-quality studies are needed to elucidate the relationship between young age and the risk of local recurrence developed within 10 years.

## Introduction

Breast cancer is a systemic malignant disease that can severely threaten a woman’s health. Because micrometastasis can be found during the early stages of this disease, comprehensive treatments including surgery, chemotherapy, hormonal therapy, radiotherapy, immunotherapy, and targeted therapy are necessary. Modified radical mastectomy with the retention of the nipple-areola complex originated in the 1970s in Europe and the United States. Now, breast-conserving therapy has become a standard treatment for stage I and stage II breast cancer in both countries, and it is also being introduced in China^1^. In breast-conserving therapy, surgeons excise the bulk of the tumor and part of the surrounding breast tissue and dissect axillary lymph nodes, and patients undergo postoperative radiotherapy to eliminate microscopic residual tumor cells.

Conserving therapy is widely preferred because it offers a better appearance, more functionality, and more psychological benefits than radical mastectomy. This type of therapy also has fewer complications and a shorter hospitalization duration than other therapies. With accumulating evidence from prospective clinical studies on breast-conserving therapy, the consensus among experts is that there are no significant differences in the local recurrence and mortality rates between conserving therapy and radical mastectomy in breast cancer patients^2–4^.

Although cases of local recurrences after breast-conserving therapy are low^5^, the development of local recurrences are of great concern. Two types of local recurrence post breast-conserving therapy have been described: true recurrence (TR) and new primary (NP) tumors. TR arises from the incomplete surgical removal of tumor cells of precancerous lesions or subclinical lesions or from malignant cells not eradicated by radiotherapy. On the other hand, NP is a different type of histological tumor or a tumor at another location (i.e., a second primary tumor). Both types of local recurrences increase the incidence of distant metastasis and mortality rate. Researchers believe that local recurrence is related to the lack of radiotherapy, positive margin, and vascular and lymphatic invasion. However, one report has also shown that local recurrence could still occur despite adequate surgical margin and radiotherapy doses^6^. Other factors, such as young age at diagnosis, could be risk factors for local recurrence in patients after breast-conserving therapy; the significance of these factors are still being debated^5–8^.

Young age is defined differently based on the epidemiological situation in different regions. For instance, young age is normally defined as age ≤ 35 years; however, in western countries, breast cancer patients younger than 40 are also considered young. The incidence of breast cancer in the United States is reportedly high in patients who are 85 years old, while the rate in patients younger than 35 years old is less than 5%. In contrast, the incidence of breast cancer increases gradually after the age of 30 and peaks at the age of 40 and 50 in the Mainland of China^9^. Similarly, in Taiwan China, patients are also younger when diagnosed with breast cancer; most are between the ages of 45 and 49^10^. As breast cancer patients in China are relatively young when compared with patients in western countries, the study of the impact of young age on the local recurrence of breast cancer after conserving therapy is of clinical significance in China.

Therefore, the present study aims to examine whether the risk of local recurrence is higher in young patients compared to old patients after breast-conserving therapy.

## Methods

This study was carried out in accordance with the PRISMA guidelines.

### Inclusion criteria

#### Participants

Subjects were eligible for inclusion if the following criteria were met: (1) subjects had primary breast cancer without both distant metastasis and other severe concurrent diseases diagnosed by clinical symptoms, physical signs, X-ray, pathology and cytology examinations; (2) underwent conserving surgery followed by standard treatment of whole breast radiation; and (3) had a minimum of 5 years follow up. There was no bias related to age, race, ethnicity, or nationality when selecting subjects.

#### Intervention

The group of younger patients was defined as the experimental group. The group of older patients was defined as control group. Young age was defined as less than 35 or 40 years old (depending on the study cited).

#### Outcome

The outcome of interest was the rate of local recurrence of breast cancer.

### Exclusion criteria

Studies were excluded according to the following criteria: (1) studies without clear diagnosis, inclusion and exclusion criteria of subjects; (2) studies with all subjects under the age of 40; (3) subjects who underwent radio- or chemotherapies before conserving therapy; (4) studies with inaccurate or incomplete data that were unable to provide outcomes; and (5) studies published repeatedly.

### Search strategy

#### Database

PubMed, EMBASE, CNKI, CBM, VIP, Wanfang, and CENTRAL were searched for studies published from database inception until July 2016.

#### Search terms

Breast tumor, breast cancer, breast neoplasms, young, age, conservation surgery, conservation treatment, local recurrence

#### PubMed search strategy

The relevant MeSH terms and keywords for PubMed literature search were as follows: (((local recurrence) AND conservative surgery) AND ((young) AND ((women) OR female))) AND ((“Breast Neoplasms”[Mesh]) OR (((((((((((((Breast Neoplasm) OR Neoplasms, Breast) OR Breast Tumors) OR Breast Tumor) OR Mammary Neoplasms, Human) OR Mammary Carcinoma, Human) OR Carcinoma, Human Mammary) OR Carcinomas, Human Mammary) OR Breast Cancer) OR Cancer of Breast) OR Mammary Cancer) OR Malignant Neoplasm of Breast) OR Malignant Tumor of Breast)).

### Literature selection

Articles were imported into EndNote software to record information such as volume and issue and the completeness of abstract. The accessibility of the articles was also checked. Irrelevant articles were excluded, and the remaining records were assessed. The articles were marked as ‘to be included,’ ‘pending,’ or ‘to be excluded (with reasons)’. For pending articles, full-text was retrieved to select studies that met the selection criteria.

### Data extraction

The following data were extracted from all of the included studies:

1. General information: Research title, authors, year of publication and published journal; and
2. Study characteristics: general information of the subjects, intervention methods, and baseline compatibility.

The processes of data selection, evaluation, and extraction were conducted by two investigators. Any discrepancies were solved by discussion or the assistance of a third investigator.

### Outcome measurements

The rates of local recurrence at 5 or 10 years after breast-conserving therapy.

### Quality assessment

The quality of the included studies was assessed using the Newcastle-Ottawa Scale (NOS). The NOS contains 8 items which are categorized into 3 broad perspectives: Selection (4 items); Comparability (1 item); and Exposure or Outcome (3 items). The NOS applies a “star” system to evaluate the quality of the included studies. A maximum of 1 star can be awarded for each item within the Selection and Exposure or Outcome categories, while a maximum of 2 stars can be assigned to Comparability. The study with the highest quality is awarded a maximum of nine stars.

### Statistical analysis

Meta-analysis was performed using Stata software version 13.1. For each included study, individual and pooled risk ratios (RR) and 95% confidence intervals (CI) were calculated. A chi-square (χ^2^) test was used to test for statistical evidence of heterogeneity between different studies, and the degree of heterogeneity was presented inI^2^ statistic. In the case of no heterogeneity (P > 0.1, I^2^ ≤ 50%), data were analyzed by a fixed-effects model. If significant heterogeneity was detected between groups (P ≤ 0.1, I^2^ > 50%), a random effects model was applied and sources of heterogeneity were evaluated through subgroup analysis. Sensitivity analysis was performed by omitting one study at a time to assess the consistency of the overall effect size.

The existence of publication bias was evaluated by the funnel plot. For the outcome measurement, the pooled effect size was used as the central axis to draw a line which intersected with x-axis vertically. The dots distributed to the left side of the axis represented the effect size that was smaller than the pooled effect size, and the dots on the other side represented the effect size that was larger than the pooled effect size. A symmetrical funnel represents the absence of bias; otherwise, publication bias exists. Asymmetry in the funnel plot was further assessed using Harbord’s modified test and Trim and Fill analysis. P value of < 0.05 was considered to be significant.

## Results

### Study selection

**Figure 1:**
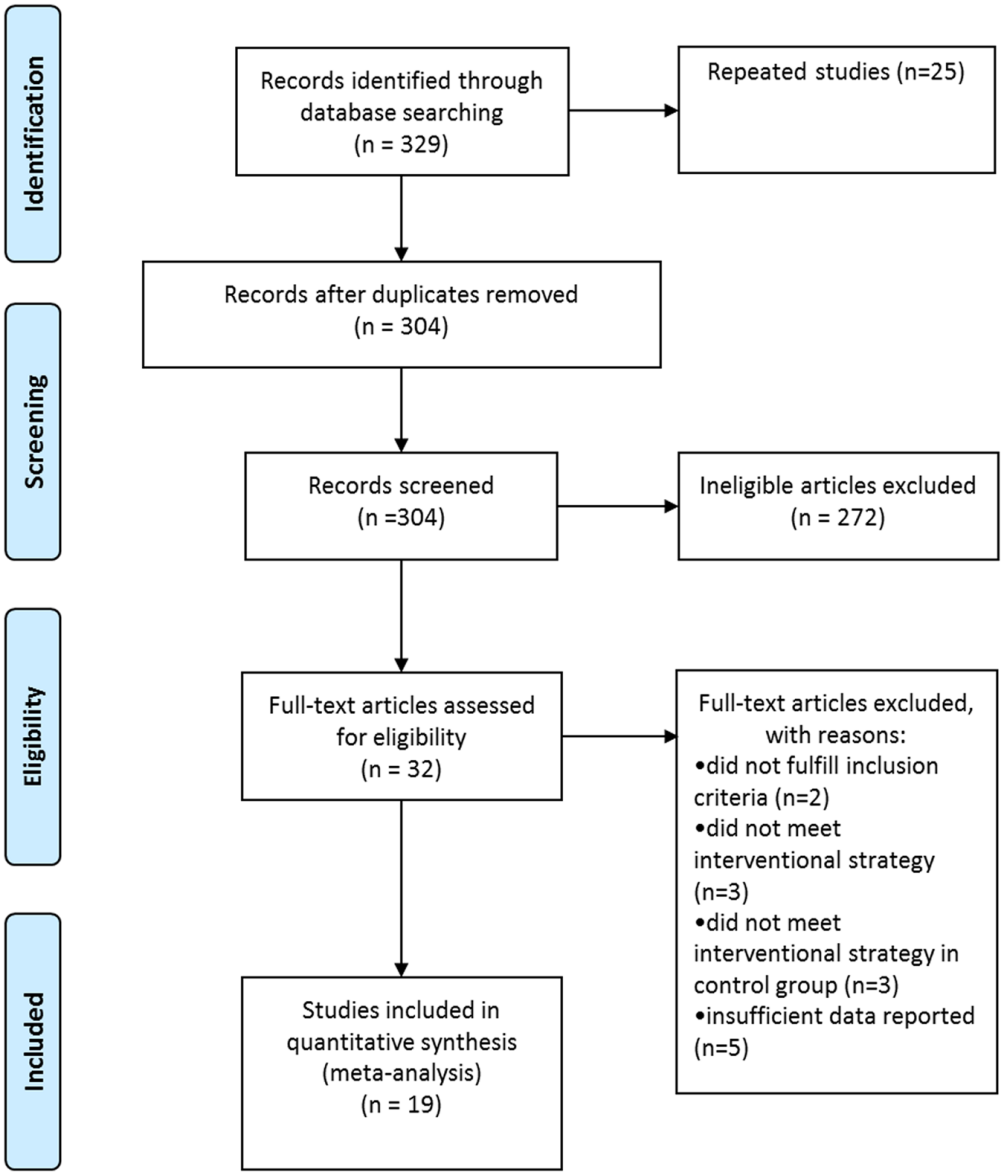
Flow diagram for selection of studies.

The search strategy yielded a total of 329 articles; of these, 25 were duplicates. The remaining 304 articles were evaluated by 2 investigators. Based on the selection criteria of subjects, intervention methods, type of research, and screening of the titles and abstracts, 272 articles were excluded, 15 were included, and 17 were listed as pending. After a full-text review of the 32 included articles and those in the pending lists, 13 articles were further eliminated. A total of 19 articles were ultimately included in the meta-analysis^7, 11–27^. All articles were in English (Fig. 1).

### Quality assessment

The included studies were assessed for risk of bias using NOS. The quality of the included studies ranges between 6–8 stars (Table 1). There were 3 studies with a score of 6 stars^15, 22, 25^, 11 with 7 stars^5, 7, 11, 12, 14, 16–18, 20, 21, 24^, and 5 with 8 stars^13, 19, 23, 26, 27^. Overall, the qualities of the included studies were high.

**Table 1:** Quality assessment of the included studies.

Study	Cohort selection				Comparability	Outcome			Score
	Representativeness of the exposed cohort	Selection of non-exposed cohort	Ascertainment of exposure	Demonstration that outcome of interest was not present at start of study	Comparability of cohorts on the basis of the design and analysis	Assessment of outcome	Follow-up was long enough for outcome to occur	Adequacy of follow up	
Clarke^11^									7
Recht^12^									7
John^13^									8
Barbara^14^									7
Burke^15^									6
Leborgne^16^									7
Pierce^17^									7
Elkhuizen^18^									7
Kini^19^									8
Bartelink^20^									7
Jobsen^21^									7
Jhingran^22^									6
Arriagada^7^									7
Ohsumi^23^									8
Bijker^24^									7
Vujovic^25^									6
E.Botteri^5^									7
Toesca^26^									8
Tovar^27^									8

### Characteristics of study selection

The following information was listed in Table 2: first author, year of publication, region, type of research, year of publication, median duration, clinical stage, 5-year local recurrence rate and 10-year local recurrence rate. All studies were published between 1985–2014, of which 7 studies were performed in North America^7, 11–14, 17, 22^, 1 in South America^27^, 9 in Europe^5, 16, 18–21, 24–26^, 1 in Asia^23^, and 1 in Australia^15^. Among the 19 included studies, 9 were prospective studies^11, 13, 15, 19, 21, 23–25, 27^, and 10 were retrospective studies^5, 7, 12, 14, 16–18, 20, 22, 26^. There are 8 studies with young defined as age ≤ 35^5, 11–15, 17, 19^, 10 studies with age ≤ 40^7, 16, 20–27^, and 1 study with age ≤ 45^18^. Only 1 study mentioned the type of recurrence detected in patients^13^.

**Table 2:** Characteristics of studies on the association between age and local recurrence of breast cancer.

Study	Country	Type of study	Age defined as young	Year the study began	Median follow-up time	Clinical stages	Young group (Experimental)		Old group (Control)	
							No. of patients with local recurrence (5 years)	No. of patients with local recurrence (10 years)	No. of patients with local recurrence (5 years)	No. of patients with local recurrence (10 years)
Clarke^11^	US	Prospective	<35	1985	5 years	Tl or T2, NO or Nl, and MO	3/32		21/424	
Recht^12^	US	Retrospective	<35	1968	63 months (range: 3–181 months)	Tl or T2	23/47		232/560	
John^13^	US	Prospective	<35	1968	80 months (range: 50–202 months)	Stage I or II	13/61 True recurrence 8/61		65/722 True recurrence 35/722	
Barbara^14^	US	Retrospective	≤35	1981	4.6years (1 month-11 years)	Stage I or II	12/38		49/683	
Burke^15^	Australia	Prospective	≤35	1992	50 months (range: 2–118 months)	Stage I or II	0/45		23/467	
Leborgne^16^	Europe	Retrospective	≤40	1973	75 months (range: 31–248 months)	Tl or T2, NO or Nl, and MO		30/83		88/730
Pierce^17^	US	Retrospective	≤35	1984	4.4 years (range: 1.0–11.5 years)	Stage I or II	3/20		12/409	
Elkhuize^18^	Netherlands	Retrospective	<45	1980	52 months (range: 1–175)	T1–2,N0–1	45/377	72/377	60/1016	189/1016
Kini^19^	England	Prospective	≤35	1980	108 months (range: 1–179 months)	Stage I or II		5/20		27/380
Bartelink^20^	Netherlands	Retrospective	≤40	1989	5.1 years (max 10.2 years)	Stage T1–2, N0–1, M0	22/221		87/2440	
Jobsen^21^	Europe	Prospective	≤40	1984	80 months (range: 3–194 months)	Stage T1	3/28		13/238	
Jhingran^22^	US	Retrospective	≤40	1980	63 months (range: 7–288 months)	Stage 0 (TisN0M0) DCIS	5/20	5/20	3/130	4/130
Arriagada2002^7^	US	Retrospective	≤40	1954	20 years (range: 0.2–9 years).	T0-T2,NO-N1,MO		28/110		48/607
Ohsumi^23^	Japan	Prospective	<40	1989	77 months (range: 1–133 months)	Tumor sizes of >3 cm	19/220		39/1333	
Bijker^24^	Netherlands	Prospective	≤40	1986	10.5 years	DCIS		23/65		184/945
Vujovic^25^	England	Prospective	≤40	1985	135 months (range: 10–224.5 months)	T1 and T2, N0	8/48	10/48	18/520	55/520
E.Botteri^5^	Europe	Retrospective	<35	2000	6 years (range: 0.2–9 years)	early-stage breast	3/113		30/2671	
Toesca^26^	Europe	Retrospective	<40	1996	80 months (range: 61–111 months)	DIN1c or DCIS G1	7/15		36/217	
Tovar^27^	Brazil	Prospective	<40	2000	10 years	—		13/89		47/731

### Correlation between age and local recurrence in women with early-stage breast cancer after breast-conserving surgery

#### Comparison of 5-year local recurrence rate between young and old patients

**Figure 2:**
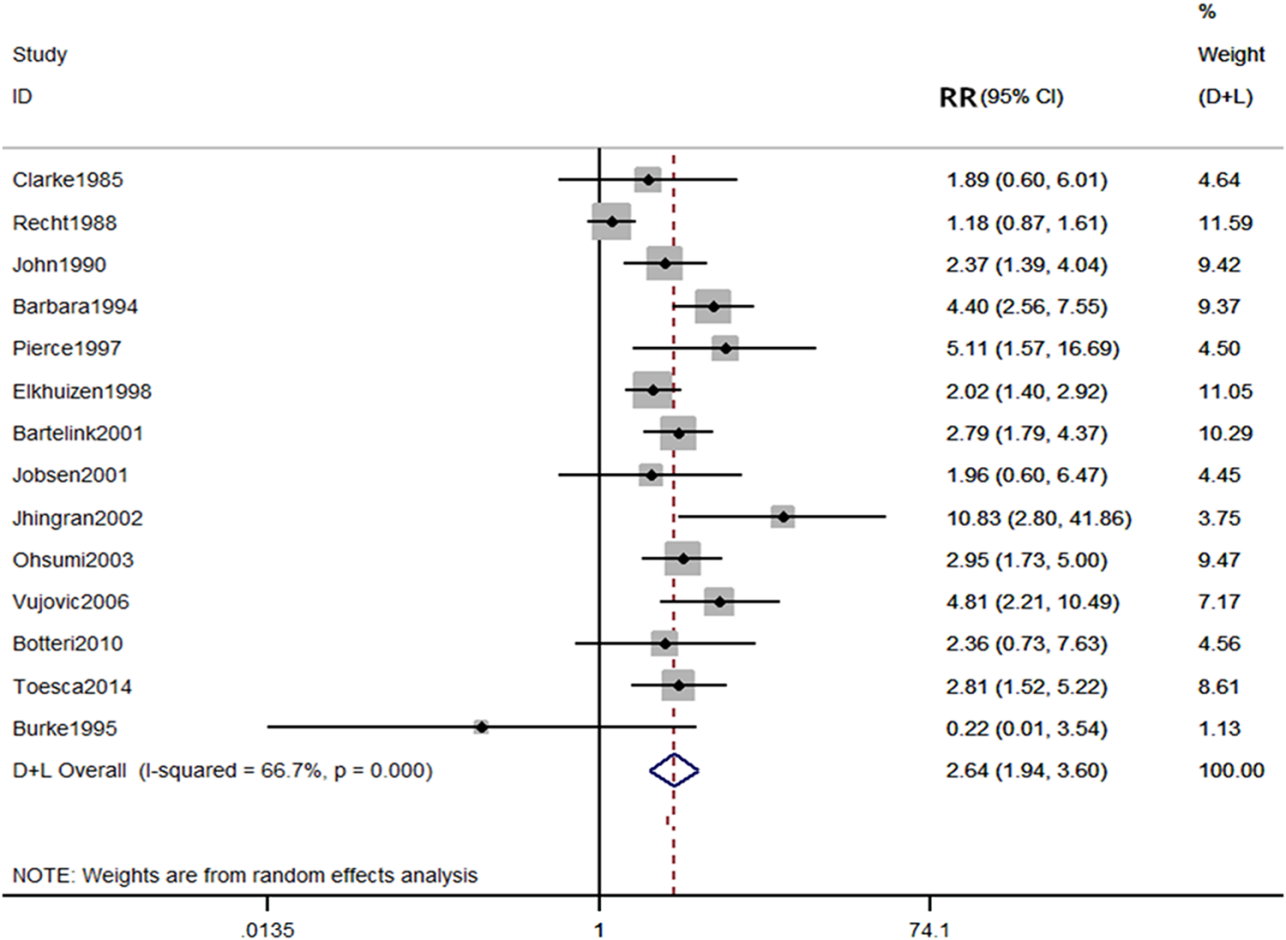
Comparison of 5-year local recurrence rate between young and old patients.

The experimental group represented young breast cancer patients who had undergone conserving therapy (young group) and the control group comprised of patients who were older than 35, 40, or 45 years (old group). Based on the 14 included studies, there were 1,285 patients in the young group and 11,830 patients in the old group^5, 11–15, 17, 18, 20, 21, 23, 25, 26^. There were 166 patients in the young group and 688 patients in the old group who experienced local occurrence within 5 years of follow-up. Heterogeneity was observed among the studies (P < 0.0001, I^2^ = 66.7%) and therefore, a random-effects model was employed to analyze the data. Our results showed a significant difference in the 5-year local recurrence rate between young and old patients (RR = 2.64, 95% CI (1.94–3.60)) (Fig. 2).

#### Comparison of 10-year local recurrence rate between the young and old patients

**Figure 3:**
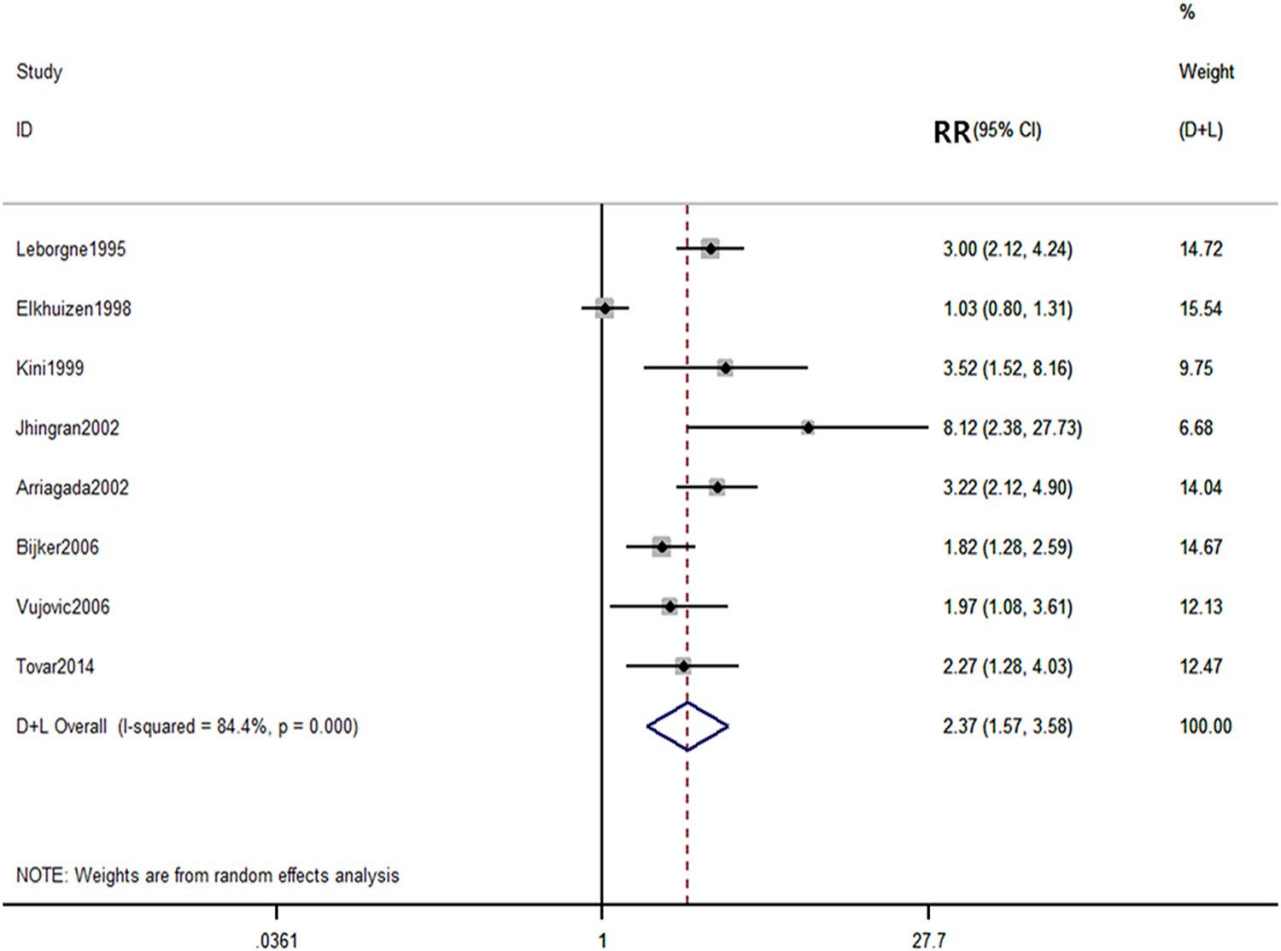
Comparison of 10-year local recurrence rate between young and old patients.

From the 8 included studies^7, 16, 18, 19, 22, 24, 25, 27^, there were 812 and 5059 patients in the young and old groups, respectively. Of these, 186 young patients and 642 old patients experienced local occurrence during the 10 years of follow up. As significant heterogeneity was observed among the studies (P < 0.0001, I^2^ = 84.8%), a random-effects model was employed to analyze the data. Our results indicated a significant difference in the 10-year local recurrence rate between the young and old patients (RR = 2.37, 95% CI (1.57–3.58) (Fig. 3).

### Subgroup analysis

#### Difference in the definition of young age

**Figure 4:**
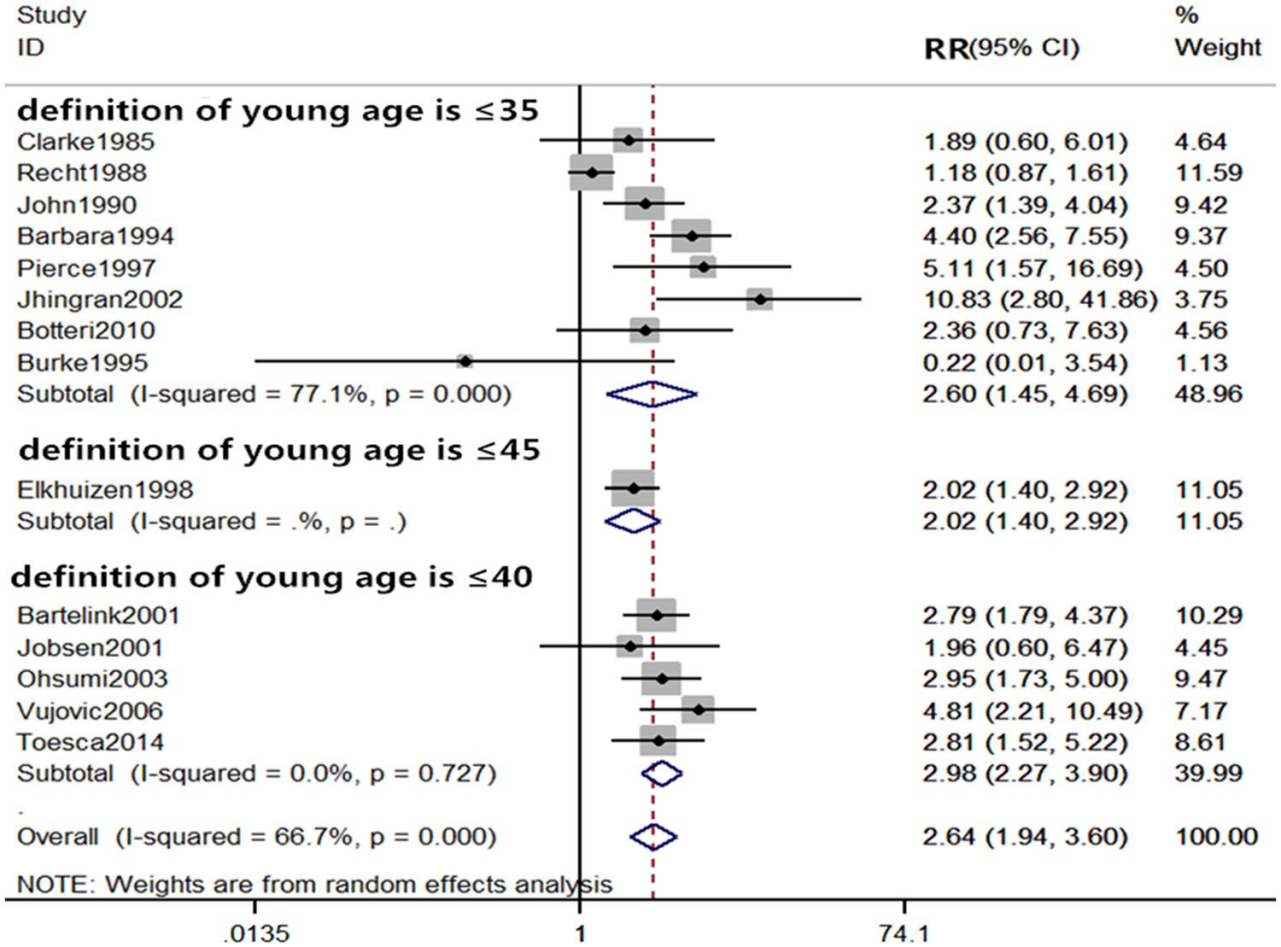
Subgroup comparison of 5-year local recurrence rate between young and old patients by definition of age.

**Figure 5:**
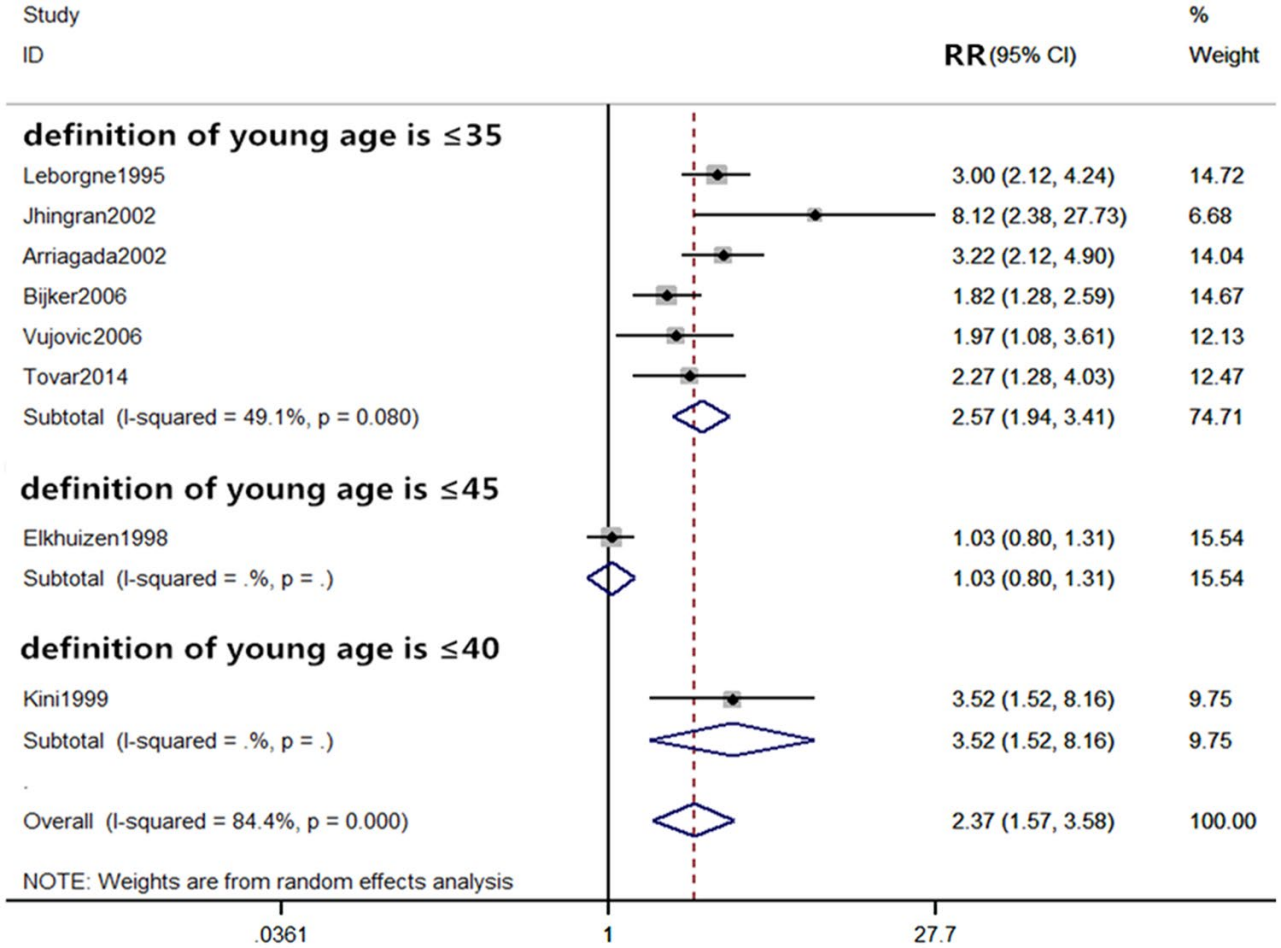
Subgroup comparison of 10-year local recurrence rate between young and old patients by definition of age.

As the statistical analyses of 5-year and 10-year local recurrence rates showed the presence of heterogeneity (I^2^ = 66.7% and I^2^ = 84.8%, respectively), we performed subgroup analyses to determine potential sources of heterogeneity. Based on the definition of young by each study, the age varies and could be stratified into three groups of ages ≤ 35, ≤ 40, and ≤ 45 for analyses. All subgroup analyses by age definitions showed significant differences in the 5-year local recurrence rate between the young and old patients (≤35 years: RR = 2.60, 95% CI (1.45–4.69); ≤ 40 years old: RR = 2.98, 95% CI (2.27–3.90); ≤ 45 years old: RR = 2.02, 95% CI (1.40–2.92)) (Fig. 4). For the analyses of 10-year local recurrence rate, results showed significant differences in the subgroups of ages ≤ 35 and ≤ 40 (RR = 3.52, 95% CI (1.52–8.16), and RR = 2.57, 95% CI (1.94–3.41) respectively), but not in the subgroup of age ≤ 45 (RR = 1.03, 95% CI (0.80–1.31)) (Fig. 5).

#### Regional differences

**Figure 6:**
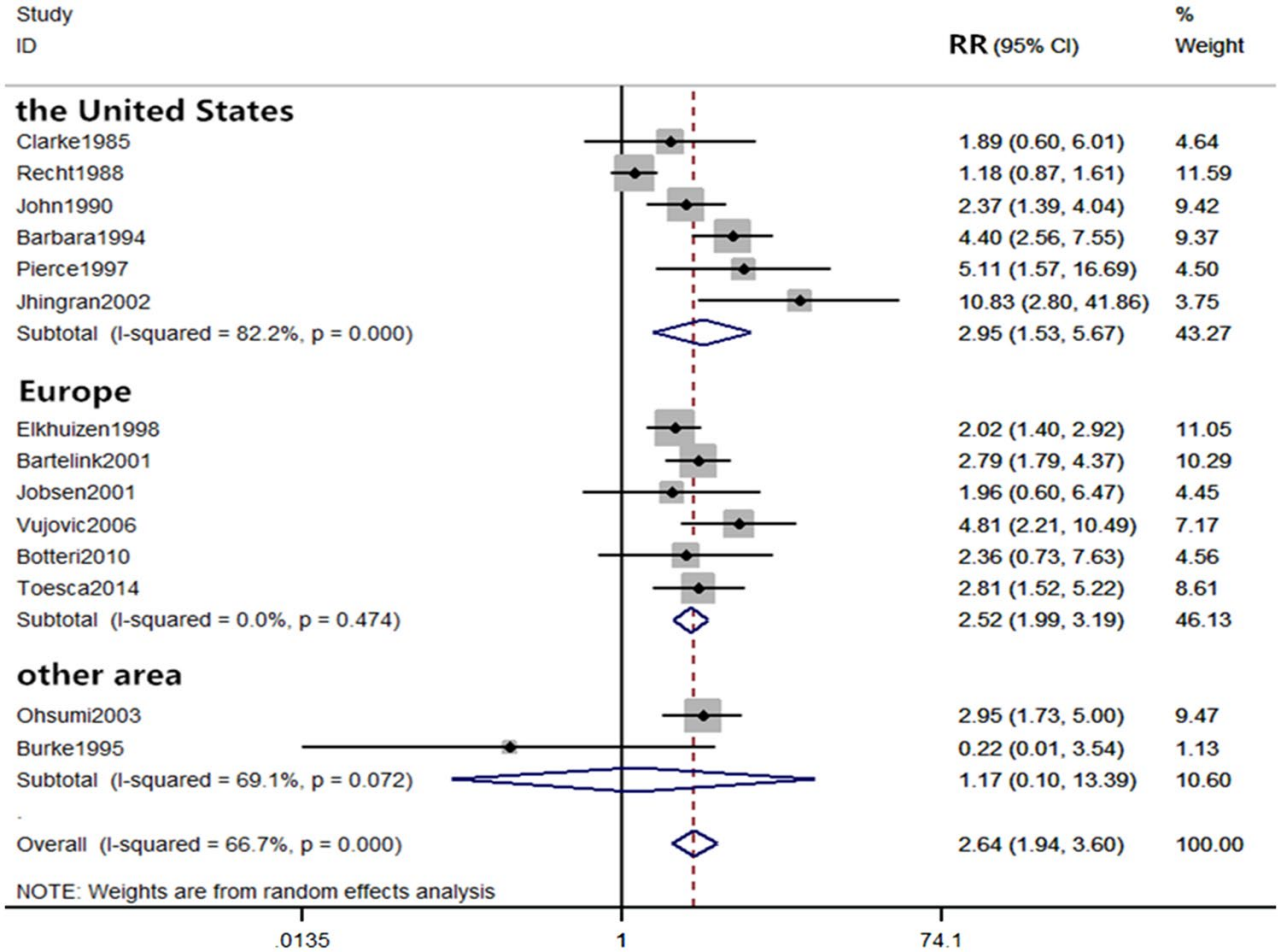
Subgroup comparison of 5-year local recurrence rate between young and old patients by countries.

**Figure 7:**
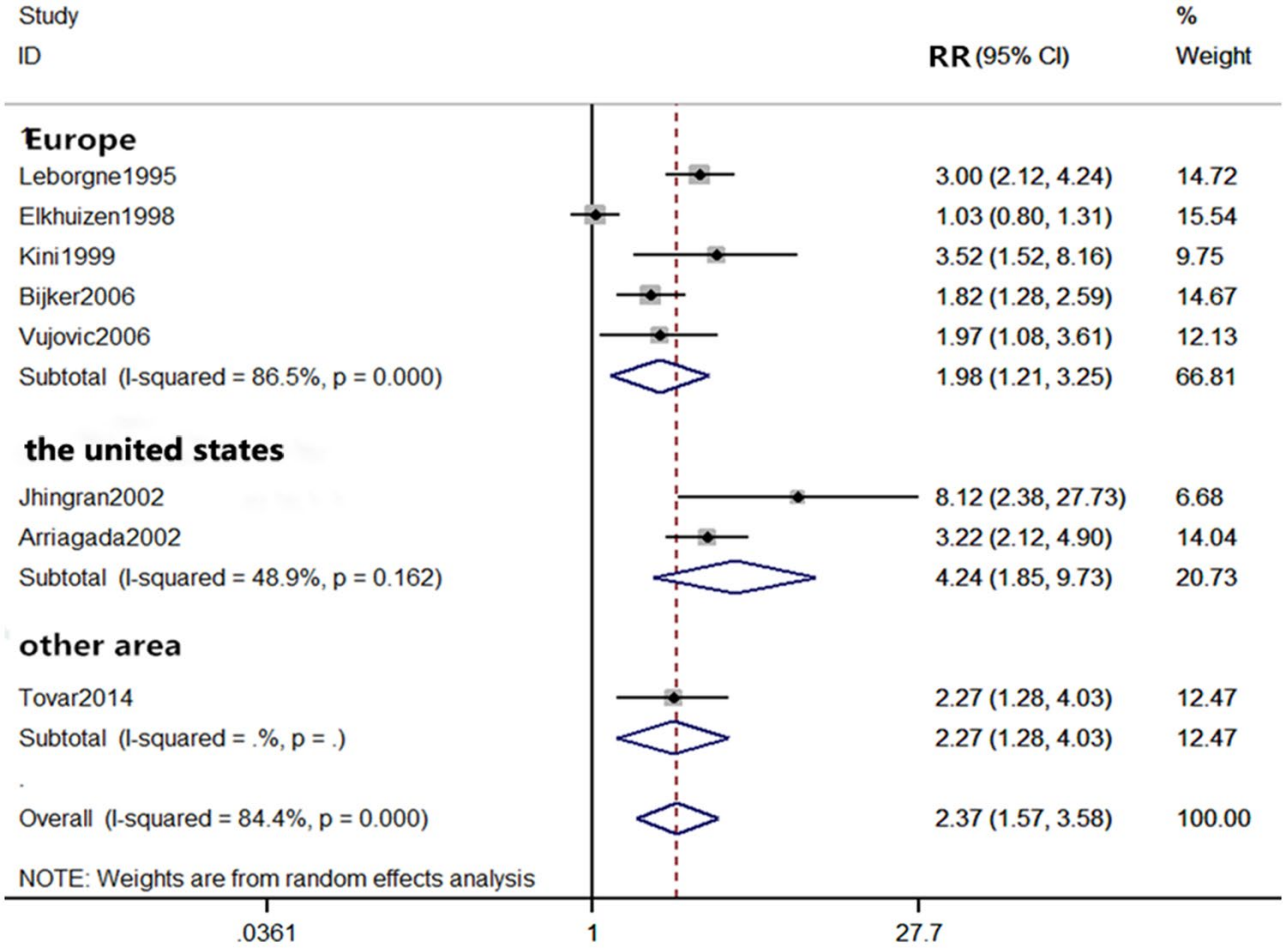
Subgroup comparison of 10-year local recurrence rate between young and old patients by countries.

The included studies were conducted in different regions and could be categorized into 3 main regions (the United States, Europe, and others) for subgroup analyses. The 5-year local recurrence rates were significantly different between the young and old in the United States and Europe (RR = 2.95, 95% CI (1.53–5.67), RR = 2.52, 95% CI (1.99–3.19), respectively), indicating that young women in the United States and Europe are more prone to local recurrence within 5 years of breast-conserving therapy than the old groups. However, pooled RRs from two studies in the subgroup of others showed no difference in the 5-year local recurrence rate (RR = 1.17, 95% CI (0.10–13.39)) (Fig. 6). Meanwhile, the 10-year local recurrence rates were significantly different in all of the subgroup analyses (the United States: RR = 4.24,95% CI (1.85–9.73); Europe: RR = 1.99,95% CI (1.20–3.29); Others: RR = 2.27,95% CI (1.28–4.03)) (Fig. 7).

### Sensitivity Analysis

**Figure 8:**
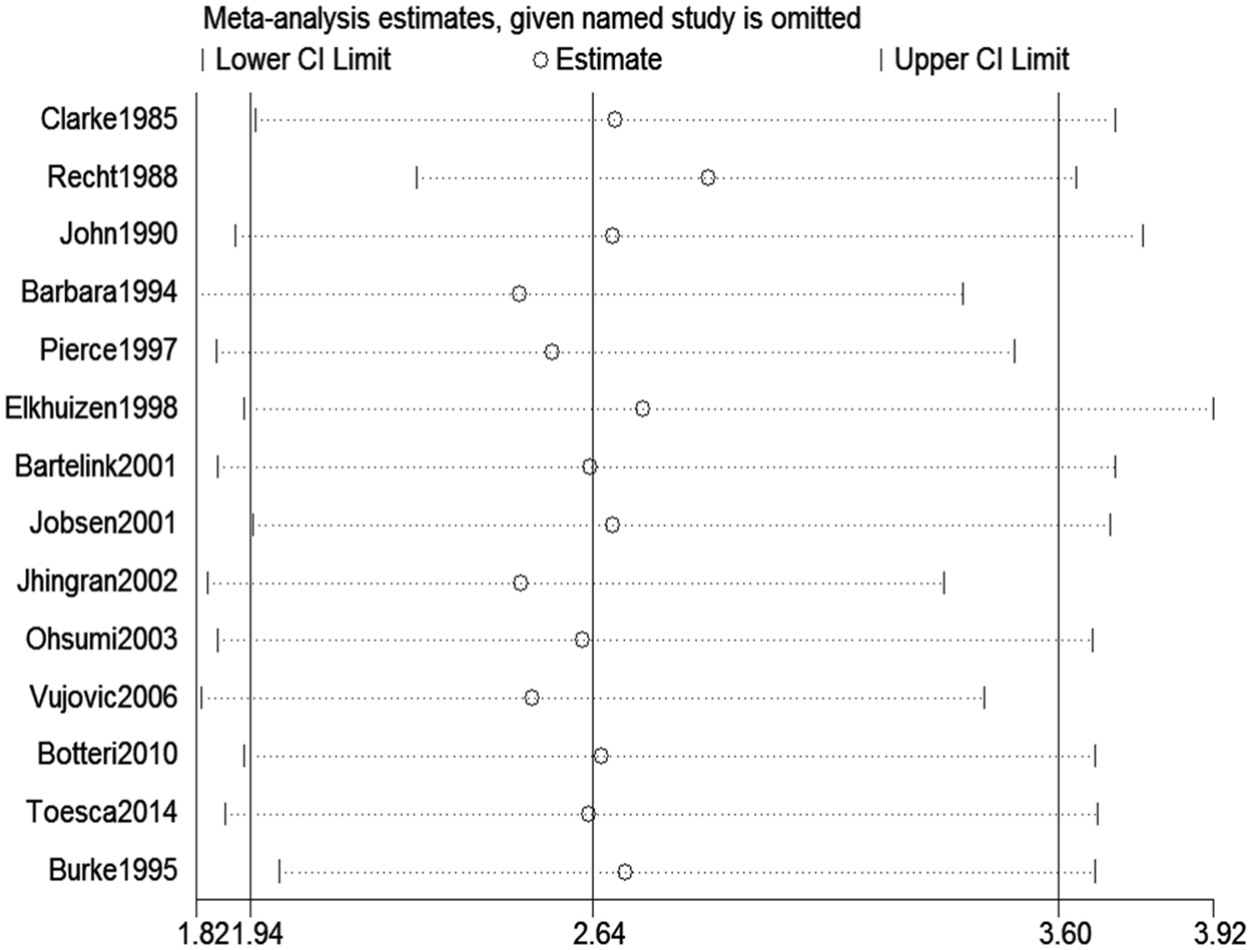
Sensitivity analysis for the 5-year local recurrence rate between young and old patients.

**Figure 9:**
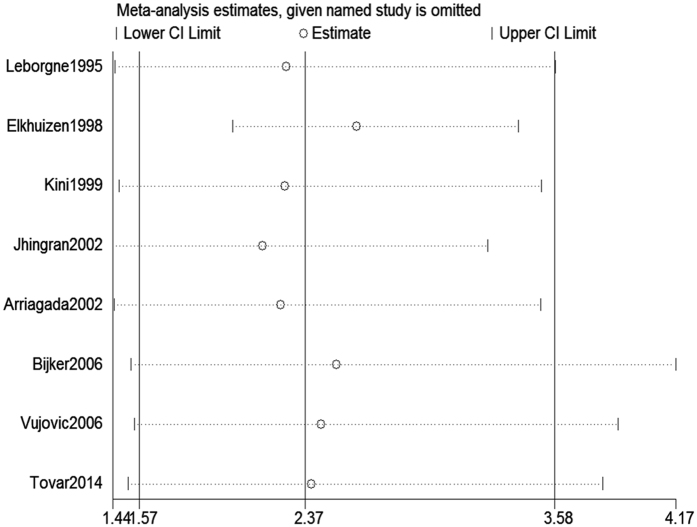
Sensitivity analysis for the 10-year local recurrence rate between young and old patients.

Sensitivity analysis is a method to test the robustness of our findings. Studies are removed one at a time and pooled RR is recalculated to determine if the overall effect size is dependent on one certain study. Our sensitivity analyses for 5- and 10-year local recurrences showed that no individual studies significantly affected the pooled RRs (Figs 8 and 9, respectively), indicating the consistency in our results.

### Publication bias

**Figure 10:**
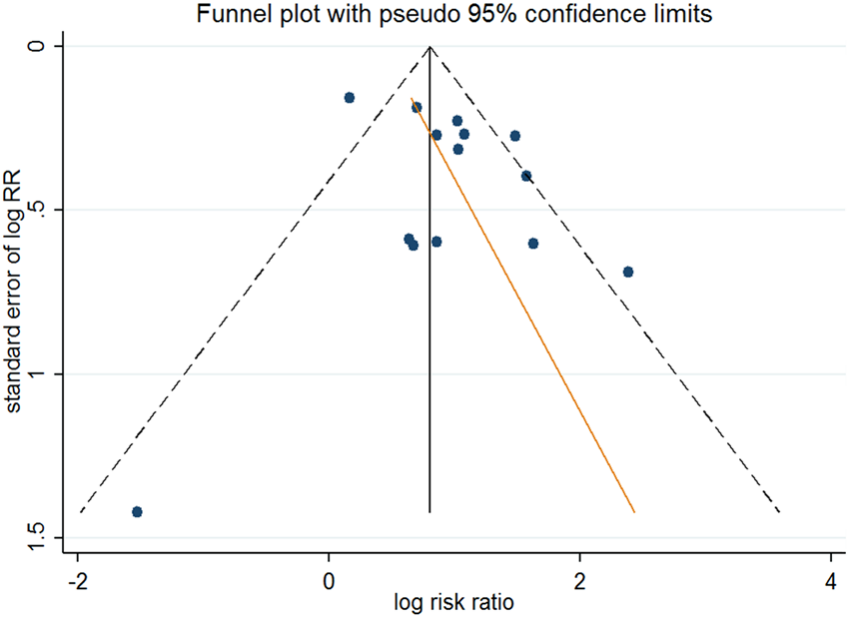
Funnel plot of the 5-year local recurrence rate.

**Figure 11:**
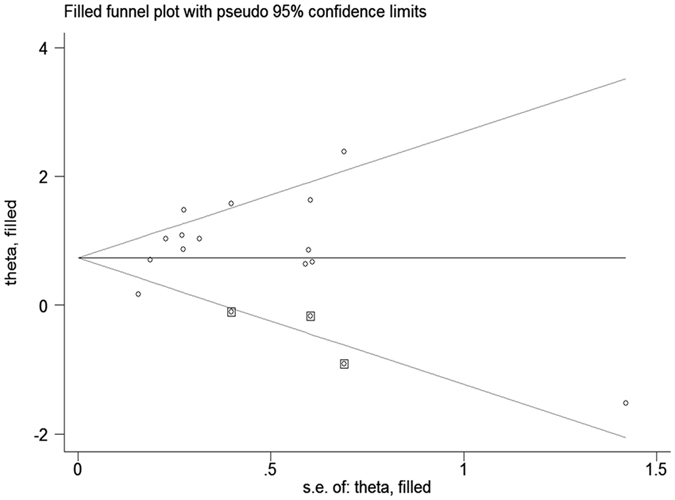
Trim and Fill analysis for the 5-year local recurrence rate. The squares represent the adjusted studies.

Publication bias was assessed by funnel plot analysis. Our result comparing the 5-year local recurrence rate between the young and old groups showed an asymmetrical funnel (Fig. 10) and Harbord’s modified test showed significant evidence of publication bias among studies (P = 0.019). By trimming and inputting 3 studies using the Trim and Fill method, the recalculated pooled RR for the 5-year local recurrence rate was 2.21, 95% CI (1.62, 3.02) (Fig. 11), which was not significantly changed from the initial estimate (RR = 2.64, 95% CI (1.94, 3.60)). Therefore, the presence of publication bias has no significant effect on the overall finding.

**Figure 12:**
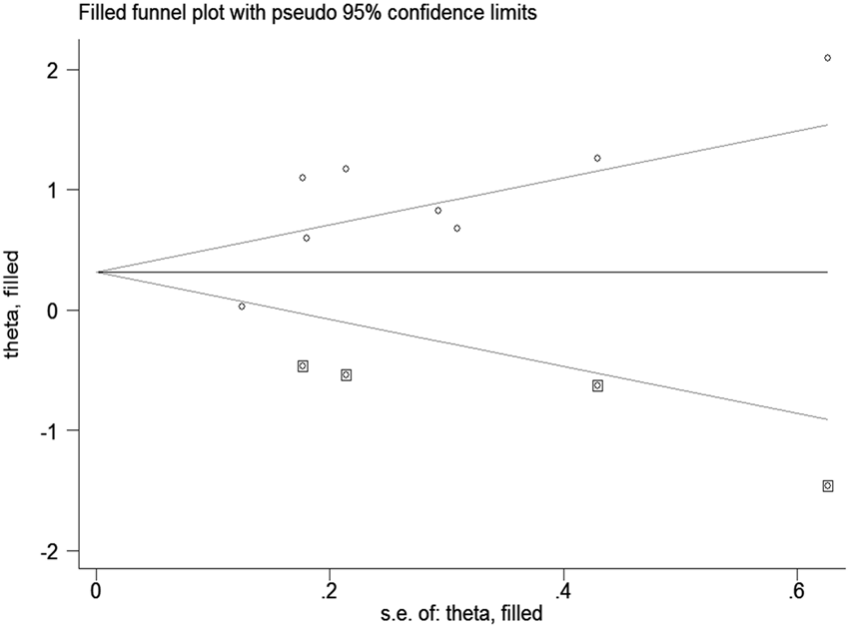
Trim and Fill analysis for the 10-year local recurrence rate. The squares represent the adjusted studies.

Because only 8 of the available studies analyzed the 10-year local recurrence rate, a funnel plot analysis was not suitable for this outcome measure. Nevertheless, Harbord’s modified test showed the presence of publication bias (P = 0.01). Using the Trim and Fill method and adjusting for 4 studies, the recalculated pooled RR for the 10-year local recurrence rate was 1.47, 95% CI (0.96, 2.27) (Fig. 12), which was significantly changed from the initial estimate (RR = 2.37, 95% CI (1.57, 3.58)). Our data suggests that there is bias in our overall finding for the 10-year local recurrence rate.

## Discussion

Our meta-analysis aims to determine whether young age is a risk factor for local recurrence in patients with early-stage breast cancer after breast-conserving therapy. We searched relevant studies published from 1966 to 2016 in databases including PubMed, EMBASE, Chinese Journal Full-text Database (CNKI), Chinese Biomedical Literature Database (CBM), VIP Chinese scientific and technical journals database (VIP), and Wanfang. Of the 329 studies found, 19 studies met our selection criteria^5, 7, 11, 13–27^. Our meta-analysis showed that young breast cancer patients are at high risk of local recurrence within 5 and 10 years after breast-conserving therapy compared to old patients (RR = 2.64, 95% CI (1.94, 3.60) and RR = 2.37, 95% CI (1.57, 3.58) respectively). Funnel plot and Harbord’s modified test showed the presence of publication bias in the analysis of 5-year local recurrence rate. Trim and Fill method showed that the impact of publication bias was within an acceptable range and did not change the overall conclusions. Publication bias also existed in the analysis of 10-year local recurrence rate. The effect size became insignificant after Trim and Fill analysis (RR = 1.47, 95% CI (0.96, 2.27). As there were only 8 studies available for the 10-year local recurrence rate analysis (relatively small sample size), Trim and Fill analysis could be inaccurate and may lead to false negative results. Our result indicates that the meta-analysis for the risk of local recurrence within 10 years of post-breast-conserving therapy is currently inconclusive and further studies are warranted.

Subgroup analyses by age definitions showed that the risk for 5-year local recurrence was significantly increased in all ≤ 35, ≤ 40, and ≤ 45 years old subgroups, while the risk for 10-year local recurrence was only significantly increased in the ≤ 35 and ≤ 40 years old subgroups. The ≤ 45 years old subgroup for the analysis of 10-year local recurrence rate showed no significant difference between the young and old groups (RR = 1.03, 95% CI (0.80, 1.31)). When young was defined as age ≤ 45, the young patients in the study by Elkhuizen *et al* ^18^. were relatively older than those in the other studies, which could lead to the insignificant result when compared to the old group. The insignificant difference observed when the age definition for the young was increased also indicates that age is a determinant for local recurrence risk. In addition, this also shows that the different definitions for young age are a source of heterogeneity in our study.

We also performed subgroup analyses on the regions where the studies were conducted, which could be categorized into the United States, Europe, and others. Our analyses showed that young age is consistently a risk factor for local recurrence in the United States and Europe. However, in the subgroup of “others,” results showed no significant difference in the 5-year local recurrence rate between the young and old patients. The pooled RR for the insignificant 5-year local recurrence result was from two studies: Ohsumi *et al*. reported a significant difference in the 5-year local recurrence rate between young and old patients in Japan^23^, while Burke *et al*. found no statistical difference between the young group and the old group in Australia^15^. Notably, in the study by Burke *et al*., the sample size for the young patients was relatively small with no incidence of local recurrence among all 45 patients (≤35 years old)^15^. Therefore, more studies are needed to validate whether young age is a risk factor for local recurrence developed within 5-year of post-therapy in regions other than the United States and Europe.

Our findings suggest that young age is a risk factor for local recurrence of breast cancer after breast-conserving therapy, which is consistent with the multivariate analysis conducted by De Bock and others in three trials in 2008^28^. Young patients may have some characteristics that promote local recurrence. A review by Anders *et al*., suggested that there is a higher percentage of ER/PR-negative tumor, HER2-positive tumor, and triple-negative tumor in young breast cancer patients compared to the old patients^29^. There were also studies which demonstrated that breast cancer patients with HER2 type tumor (ER/PR−, HER2+) and basal type tumor (triple negative, ER/PR−, HER2−) displayed increased risk for local recurrence^30–32^. Therefore, compared to older patients, the tumor subtypes susceptible to local recurrence are relatively higher in young breast cancer patients. Besides that, breast cancer in young patients is also more likely to be caused by familial mutations^33^. Hereditary familial breast cancer tends to have an early onset, particularly in those with BRCA1/BRCA2 mutations^33^. This type of breast cancer is also more aggressive, and patients tend to relapse after therapies.

This study has a few limitations. Studies included in this meta-analysis are published studies. The results that achieved statistical significance have a greater chance to be reported or published when compared to those results that are invalid or failed to achieve statistical significance. This phenomenon will exaggerate the effect of the group tested. We also did not analyze the effects of other age-related factors, such as the levels of estrogen, progesterone, progesterone receptor, and HER2 receptor, on local recurrence. These factors could be included in future studies for a more convincing result.

With the increasing rate of breast cancer in young patients, especially in Asia, and the significantly higher rate of local recurrences in young patients compared to old patients, young patients should be treated more cautiously. Young patients should be given a variety of auxiliary treatments, which include standardized radiotherapy, chemotherapy, hormonal therapy, and biological therapy after conserving surgery. In addition, more studies should aim to reveal the mechanisms underlying the pathogenesis of early-stage breast cancer in young patients and to improve personalized treatment. Our study found a strong association between young age and 5-year local recurrence rate in patients with early-stage breast cancer after breast-conserving therapy. Future high-quality studies are needed to determine the association of young age with the risk of local recurrence developed within 10 years after breast-conserving surgery.

In the subgroup analysis of regional differences (Fig. 6), we showed that young women in the United States and Europe are more prone to local recurrence within 5 years of breast-conserving therapy than older women when compared to women from a collection of “other” regions, which included Brazil, Japan, and Australia. However, these data do not directly imply that young women from United States and Europe have higher risk of local recurrence when compared to young women from other continents. One reason we obtained statistical significance in the women from United States and Europe but not in the women from other regions is that the present meta-analysis includes more studies from United States and Europe than other regions. When analyzing the local recurrence within 5 years of breast-conserving therapy, the present meta-analysis includes 6 studies from the United States and 6 studies from Europe, but only 2 studies from others regions. Therefore, it is invalid to claim that young women from other continents have lower risk of 5-year local recurrence than women from United States and Europe. The data suggest that more high-quality studies from other countries/regions are needed to confirm whether the higher risk of local recurrence exists in young women from different geographical regions and ethnicities.

## Conclusions

In conclusion, young age is a significant risk factor for local recurrence in women with early-stage breast cancer after breast-conserving therapy. Hence, a different approach should be considered when treating young patients due to their unique breast cancer features.
